# The Monoheme *c* Subunit of Respiratory Alternative Complex III Is Not Essential for Electron Transfer to Cytochrome *aa*_3_ in Flavobacterium johnsoniae

**DOI:** 10.1128/spectrum.00135-21

**Published:** 2021-06-30

**Authors:** Katarzyna Lorencik, Robert Ekiert, Yongtao Zhu, Mark J. McBride, Robert B. Gennis, Marcin Sarewicz, Artur Osyczka

**Affiliations:** a Department of Molecular Biophysics, Faculty of Biochemistry, Biophysics and Biotechnology, Jagiellonian University, Kraków, Poland; b Department of Biological Sciences, Minnesota State University Mankato, Mankato, Minnesota, USA; c Department of Biological Sciences, University of Wisconsin–Milwaukee, Milwaukee, Wisconsin, USA; d Department of Biochemistry, University of Illinois at Urbana-Champaign, Urbana, Illinois, USA; University of Minnesota

**Keywords:** *Flavobacterium johnsoniae*, alternative complex III, quinone reductase, menaquinol, oxygen consumption, *ompA* promoter

## Abstract

Bacterial alternative complex III (ACIII) catalyzes menaquinol (MKH_2_) oxidation, presumably fulfilling the role of cytochromes *bc*_1_/*b*_6_*f* in organisms that lack these enzymes. The molecular mechanism of ACIII is unknown and so far the complex has remained inaccessible for genetic modifications. The recently solved cryo-electron microscopy (cryo-EM) structures of ACIII from Flavobacterium johnsoniae, Rhodothermus marinus, and Roseiflexus castenholzii revealed no structural similarity to *cytochrome bc_1_*/*b*_6_*f* and there were variations in the heme-containing subunits ActA and ActE. These data implicated intriguing alternative electron transfer paths connecting ACIII with its redox partner, and left the contributions of ActE and the terminal domain of ActA to the catalytic mechanism unclear. Here, we report genetic deletion and complementation of *F. johnsoniae actA* and *actE* and the functional implications of such modifications. Deletion of *actA* led to the loss of activity of cytochrome *aa_3_* (a redox partner of ACIII in this bacterium), which confirmed that ACIII is the sole source of electrons for this complex. Deletion of *actE* did not impair the activity of cytochrome *aa_3_*, revealing that ActE is not required for electron transfer between ACIII and cytochrome *aa_3_*. Nevertheless, absence of ActE negatively impacted the cell growth rate, pointing toward another, yet unidentified, function of this subunit. Possible explanations for these observations, including a proposal of a split in electron paths at the ActA/ActE interface, are discussed. The described system for genetic manipulations in *F. johnsoniae* ACIII offers new tools for studying the molecular mechanism of operation of this enzyme.

**IMPORTANCE** Energy conversion is a fundamental process of all organisms, realized by specialized protein complexes, one of which is alternative complex III (ACIII). ACIII is a functional analogue of well-known mitochondrial complex III, but operates according to a different, still unknown mechanism. To understand how ACIII interacts functionally with its protein partners, we developed a genetic system to mutate the Flavobacterium johnsoniae genes encoding ACIII subunits. Deletion and complementation of heme-containing subunits revealed that ACIII is the sole source of electrons for cytochrome *aa_3_* and that one of the redox-active subunits (ActE) is dispensable for electron transfer between these complexes. This study sheds light on the operation of the supercomplex of ACIII and cytochrome *aa_3_* and suggests a division in the electron path within ACIII. It also shows a way to manipulate protein expression levels for application in other members of the *Bacteroidetes* phylum.

## INTRODUCTION

Respiratory energy conversion, which is a fundamental process of all organisms, is realized by specialized protein complexes that form electron transfer chains. The common feature of most of these chains is the presence of a ubiquinol-cytochrome *c* oxidoreductase (mitochondrial complex III; cytochrome *bc_1_*), or a plastoquinol-plastocyanin oxidoreductase (cytochrome *b*_6_*f*). Some species of bacteria have genes encoding the terminal oxidase but lack genes encoding obvious components of an analogue of mitochondrial complex III. The search for an enzyme that could fulfil the role of the absent cytochrome *bc_1/_b*_6_*f* in such bacteria led to the discovery of alternative complex III (ACIII) in Chloroflexus aurantiacus (1, 2) and in Rhodothermus marinus (3, 4). Genes encoding the components of ACIII were later found in many other bacteria belonging to diverse phyla (5). ACIII is thought to function similarly to cytochrome *bc_1_* by transferring electrons from the membrane-soluble quinone pool to the terminal oxidase, and in the process contributes to the proton motive force required for ATP synthesis.

Recently, ACIII structures from three species, Flavobacterium johnsoniae (PDB: 6BTM), *R. marinus* (PDB: 6F0K), and Roseiflexus castenholzii (PDB: 6LOE and 6LOD) were solved by cryo-EM, revealing no structural similarity to cytochrome *bc_1_*/*b*_6_*f* (6–8). Based on the sequence similarities, ACIII is considered to be a new member of the complex iron-sulfur molybdoenzyme (CISM) superfamily, but without the molybdenum cofactor (2, 5).

**FIG 1:**
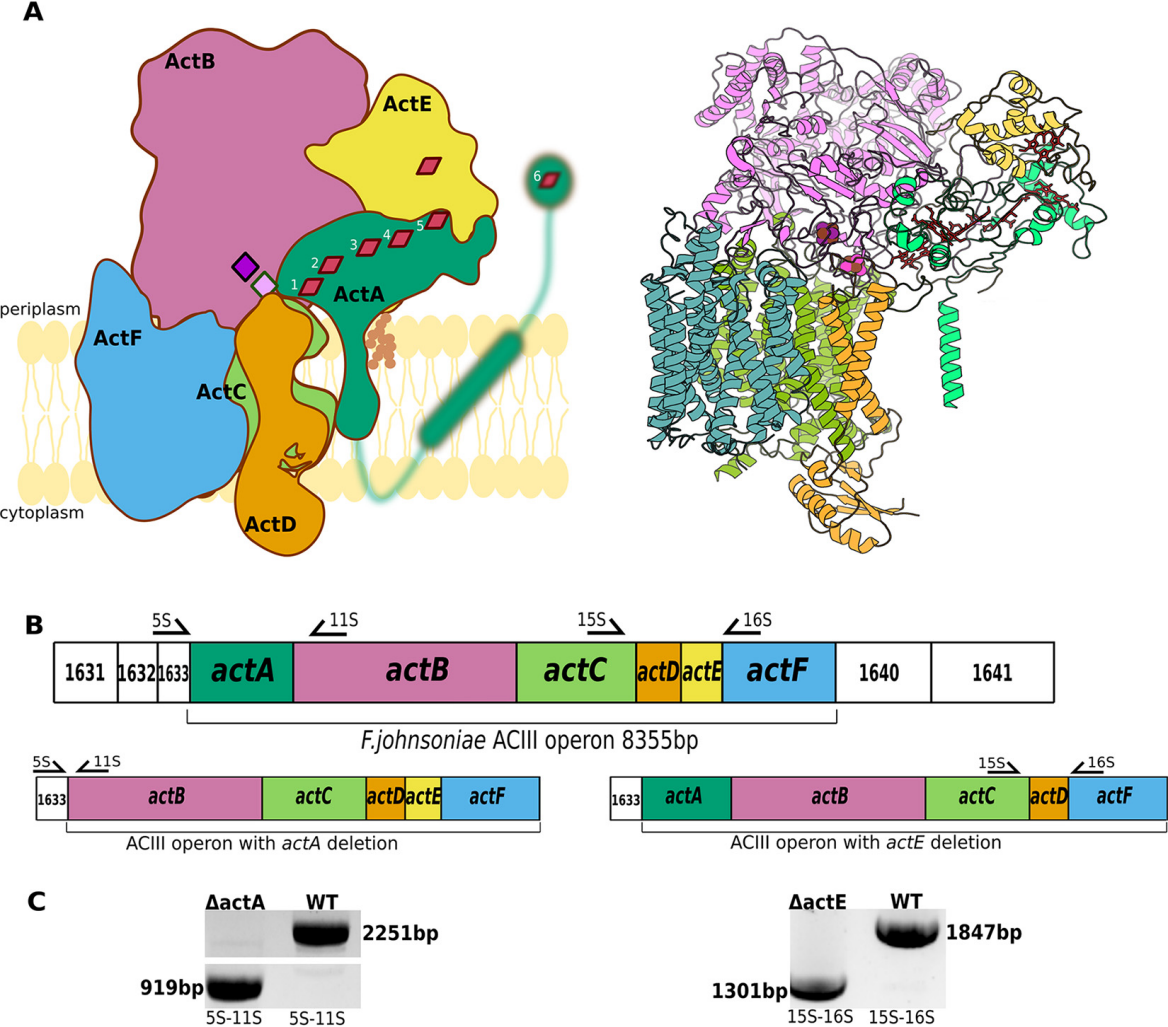
*F. johnsoniae* ACIII complex composition and deletions of *actA* and *actE*. (A, left) Schematic representation of the ACIII complex subunits. Red rhombuses represent heme *c* molecules and pink and purple diamonds depict [3Fe-4S] and [4Fe-4S] clusters, respectively. The ActE lipid anchor is depicted as brown balls; the ActB lipid anchor is not shown. The blurred green represents an N-terminal domain of ActA that was not resolved by cryo-EM. (A, right) Ribbon representation of ACIII cryo-EM structure (PDB: 6BTM) (B) Genetic organization of the ACIII operon with marked primers used for testing of *actA* and *actE* deletions. The numbers refer to *F. johnsoniae* gene loci (for example, 1631 stands for Fjoh_1631). (C) Results of colony PCRs confirming deletions of *actA* and *actE* from the *F. johnsoniae* genome. Amplification primers are listed beneath the lanes.

*F. johnsoniae* ACIII is composed of six subunits, namely, ActA through ActF (Fig. 1A). ActA accommodates six *c-*type hemes. ActB is the largest subunit of the complex and consists of two domains, B1 and B2. Although the B2 domain possesses binding motifs for four iron-sulfur clusters, only two iron-sulfur clusters are observed in the cryo-EM map. The ActC and ActF sequences each have a motif that is predicted to bind quinol (9). From the structural studies, ActC emerges as the subunit where quinol oxidation takes place, but quinol was not directly observed in the cryo-EM map. The transmembrane ActD has no cofactors and its function remains unknown. ActE is a membrane-anchored subunit that contains one heme *c* (6). *R. marinus* and *R. castenholzii* ACIII structures are highly similar, with the exception that the ActA subunit lacks one of the hemes (7, 8) that is present in *F. johnsoniae*. Interestingly, unlike in *R. castenholzii*, ACIII from *F. johnsoniae* and *R. marinus* was isolated in a complex with an *aa*_3_-type oxygen reductase (cytochrome *aa_3_* in *F. johnsoniae* and cytochrome *caa_3_* in *R. marinus*) and it was in these forms that they were analyzed by cryo-EM. The tight association of ACIII with *aa*_3_-type oxygen reductase suggested that the two complexes might form a supercomplex (6, 7), similar to the supercomplexes described in other electron transport systems (10–12).

The catalytic mechanism of ACIII is unknown. Furthermore, considering the architecture of cofactor chains, it is clear that it is different from that of canonical cytochrome *bc_1_*. Available spectroscopic and enzymatic data are limited and concern mostly ACIII from *R. marinus* (3, 13–15). In this context, the three cryo-EM structures provided important structural insights with which the first mechanistic models were formulated. They propose that menaquinol (MKH_2_), which is a natural ACIII substrate (16, 17), is oxidized at the quinone-binding site located presumably near the periplasmic side of ActC and the two electrons are transferred sequentially through the nearby [3Fe-4S] cluster of ActB to a wire composed of five hemes *c* from ActA and then to heme *c* of ActE.

Despite the postulated role of the [3Fe-4S] cluster in oxidation of MKH_2_, the role of all iron-sulfur clusters remains unclear. Part of the problem is inconsistencies of cryo-EM structures with empirical spectroscopic data.

While the amino acid sequence of ActB implicates the presence of the binding motifs for four iron-sulfur clusters, only two of them were identified in the cryo-EM structure of *F. johnsoniae* ACIII: the previously mentioned [3Fe-4S] cluster and a [4Fe-4S] cluster adjacent to it (6). For the *R. marinus* and *R. castenholzii* ACIII complexes, all clusters were present in the structure (i.e., one [3Fe-4S] and three [4Fe-4S]), whereas the electron paramagnetic resonance (EPR) measurements identified the spectral features of only one of them (the [3Fe-4S] cluster) for *R. marinus* (3).

The positions of these four clusters implied that, if active, they would direct electron transfer away from the chain of hemes. For *R. marinus*, it was hypothesized that the chain of four iron-sulfur clusters forms another electron transfer wire to a yet-unknown periplasmic acceptor. Alternatively, this wire could be an electron input chain allowing an alternate source of electrons from the periplasm (7). On the contrary, for *R. castenholzii*, it was postulated that the [4Fe-4S] clusters form a dead end. These clusters may serve as an electron sink limiting formation of reactive oxygen species (8). However, the actual roles of these clusters remain unknown.

The final steps of electron transfer from heme *c* to the electron-accepting partner of ACIII differ in the three organisms. *R. castenholzii* ACIII is engaged in cyclic electron transfer during photosynthesis. Electrons from ActE are transferred to the small blue copper protein auracyanin, and then to the photosynthetic reaction center (8).

For *F. johnsoniae* ACIII, it was proposed that the two final cofactors of ACIII involved in electron transfer to the cytochrome *aa_3_* are the heme *c* of ActE and the N-terminal sixth heme *c* of ActA. The latter heme is located in what appears to be a membrane-anchored, mobile globular domain. It was thus suggested that ActE receives electrons from the five-heme “electron wire” in ActA and then donates them to the heme *c* in the N-terminal ActA mobile domain. This ActA domain moves between two positions, shuttling electrons directly from ActE to cytochrome *aa_3_* (6).

For *R. marinus*, due to the lack of the N-terminal mobile domain of ActA containing the sixth heme *c*, it was postulated that ActE accepts electron from the hemes *c* chain of ActA and then serves as the direct electron donor to cytochrome *caa_3_* (7).

In view of new structural information on ACIII, several mechanistic concepts of operation of this enzyme emerged. However, whether ActE transfers electrons to the terminal oxidase remained an open question. Addressing this and other questions was severely limited due to the lack of a system to genetically modify this protein complex. Here, we report such a system. Modification of the genetic tools used to manipulate *F. johnsoniae* and related bacteria (18) enabled us to delete and complement the genes encoding the ActA and ActE subunits. The study revealed that ActE is not essential for electron transfer to cytochrome *aa_3_*. The electron transfer from ACIII to cytochrome *aa_3_* may occur directly from ActA. However, *in vivo* analysis of the *actE* deletion mutant revealed that this subunit is beneficial for bacterial growth. The possible electron paths involving ActE are discussed.

## RESULTS AND DISCUSSION

### Deletion of *actA* and *actE* genes inhibits bacterial growth.

*F. johnsoniae* seemed suitable for ACIII genetic deletion for two reasons. First, ACIII was predicted to be dispensable for growth because of the presence of another type of quinol oxidase*, bd* oxidase (Fjoh_4878, Fjoh_4879), that is predicted to transfer electrons directly from the quinone pool to oxygen, bypassing ACIII and cytochrome *aa*_3_. Therefore, a strain lacking ACIII might still grow under aerobic conditions using oxygen as a terminal electron acceptor. Second, tools to genetically manipulate *F. johnsoniae* are available (18, 19).

Given that the plasmid-based expression of the entire 8,400-bp ACIII operon (Fig. 1B) seemed unfeasible, we targeted single ACIII components for expression individually. We chose the heme-containing subunits ActA and ActE, which are implicated in the catalytic mechanism and are convenient to monitor by optical spectroscopy and gel electrophoresis followed by heme staining. This allowed us to biochemically verify deletion and complementation of the genes encoding these ACIII complex proteins.

**FIG 2:**
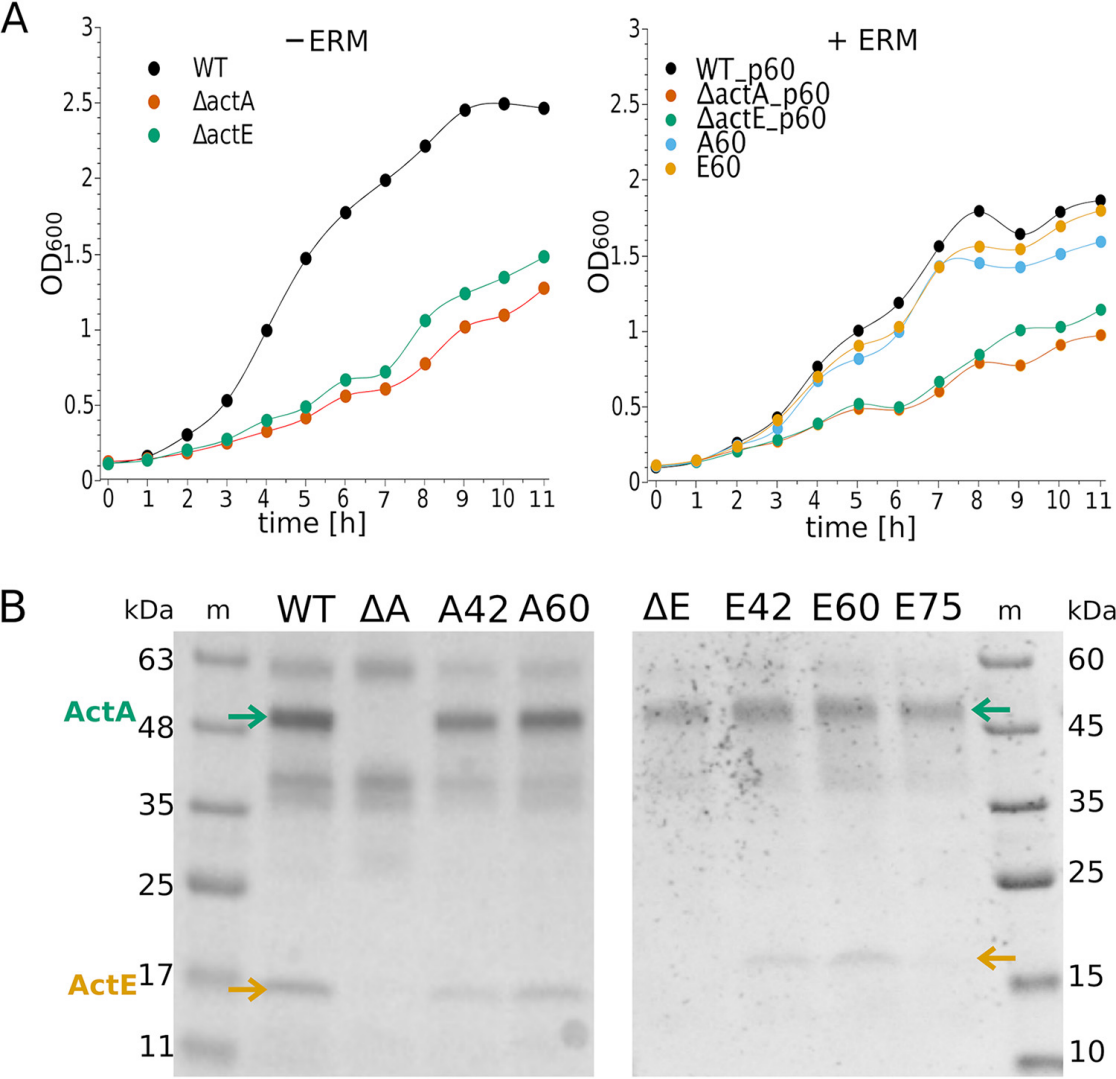
Effects of *actA* and *actE* deletion and complementation on bacterial growth and expression of heme *c*-containing proteins. (A) Growth curves of Δ*actA* and Δ*actE* (left) and of the complemented mutants (right) in comparison to WT; +ERM indicates culture medium with erythromycin. Data points were connected with splines for better visibility. The panels show representative growth curves of 4 (left) and 3 (right) independent experiments. p60, empty vector pCP11_60%; A60, Δ*actA* complemented with pCP11_A60; E60, Δ*actE* complemented with pCP11_E60. (B) TMBZ staining of heme *c* of the membrane samples on SDS-PAGE gels; m, protein marker; ΔA, Δ*actA* strain; and ΔE, Δ*actE* strain. A42 and A60 refer to Δ*actA* complemented with pCP11_A42 and pCP11_A60, respectively. E42, E60, and E75 refer to Δ*actE* complemented with pCP11_E42, pCP11_E60, and pCP11_E75, respectively. Green and orange arrows indicate the ActA and ActE subunit, respectively. Unmarked bands are not specific for ACIII complex and represent other heme *c*-containing proteins present in the membranes.

Using the allelic exchange technique developed previously to study *F. johnsoniae* and related bacteria (18), we successfully deleted the entire *actA* gene (Δ*actA*) and *actE* gene (Δ*actE*) individually from the *F. johnsoniae* genome (Fig. S1 in the supplemental material). Colony PCR and DNA sequencing confirmed the deletions of the respective genes (Fig. 1C). As expected, the deletions were not lethal and both the Δ*actA* and Δ*actE* mutants grew, although less well than did the wild-type strain (Fig. 2A, left). The decreased growth rates of the mutants suggest that the absence of either ActA or ActE leads to decreased overall efficiency of the associated electron transport chains in securing the energy demands of the cells.

The deletions of *actA* and *actE* (and their respective complemented strains, see next paragraph) were also confirmed at the protein level in the membrane samples using the 3,3′,5,5′-tetramethyl benzidine (TMBZ) gel-staining method that selectively detects only proteins with covalently attached hemes. In Δ*actA* and Δ*actE* strains, ActA (green arrow) and ActE (orange arrow) were missing, respectively (Fig. 2B). Interestingly, in the Δ*actA* strain, ActE was also missing. This indicates that incorporation of ActE into the membrane and its assembly with other subunits of ACIII requires the presence of ActA. Considering the structure of ACIII, it can be speculated that the missing docking area on ActA for ActE prevents ActE from interacting with other subunits of ACIII. This occurs despite the fact that ActE contains a lipid anchor, which should allow membrane incorporation of this subunit. In contrast, ActA did not require the presence of ActE to be stably incorporated into the membrane.

### Plasmid-based expression of *actA* and *actE* requires adjustment of promoter activity.

**FIG 3:**
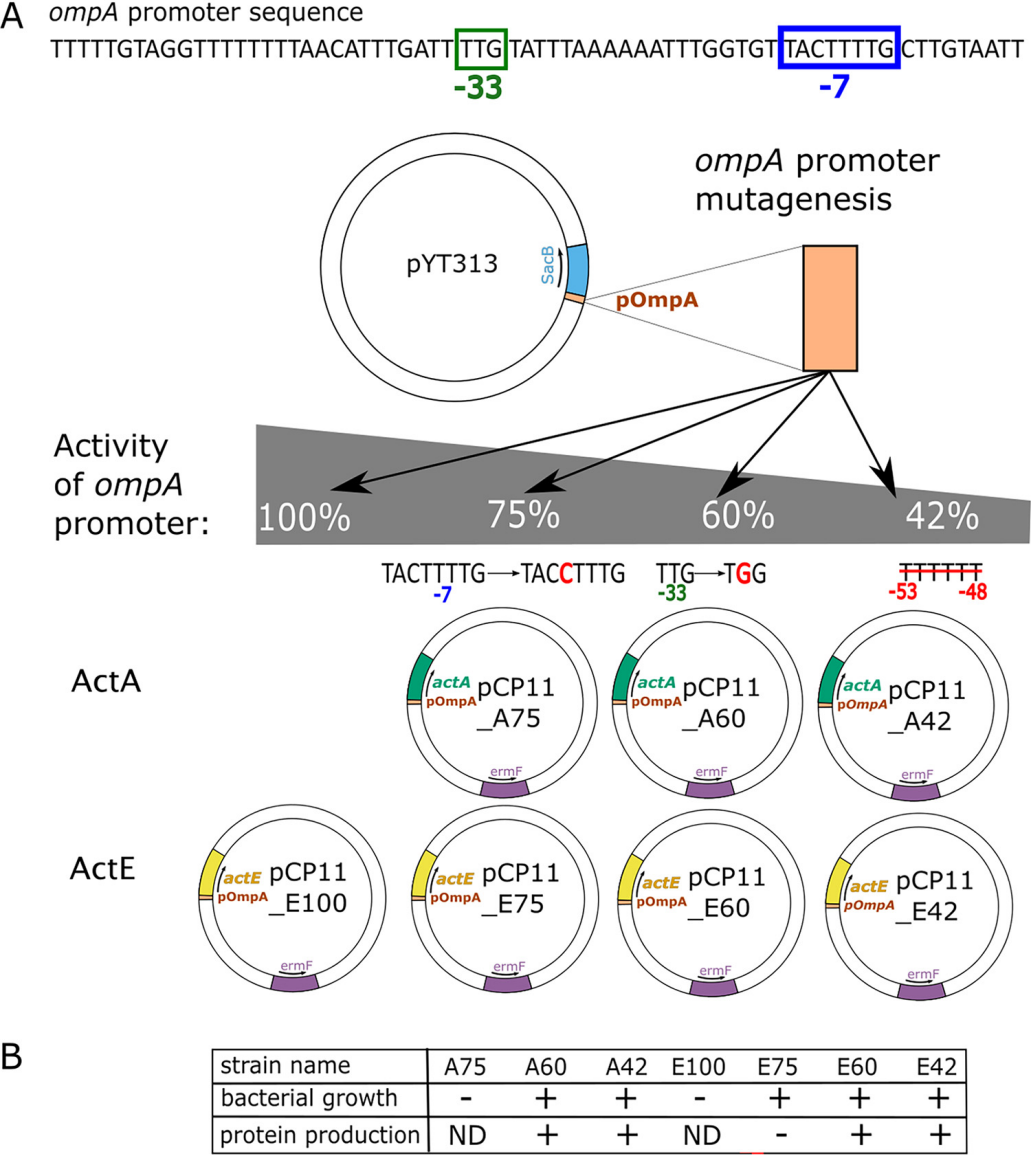
Mutagenesis of the *ompA* promoter. (A) *ompA* promoter (pOmpA) was amplified from pYT313, PCR-mutagenized, and inserted into the pCP11 plasmid in front of the *actA* or *actE* gene, generating a series of plasmids with different promoter activities of 42%, 60%, 75%, or 100% (without mutation). The plasmid nomenclature is explained in the text. ermF, erythromycin resistance gene. (B) The effects of various versions of the *ompA* promoter preceding *actA* and *actE* genes on bacterial growth and protein production; ND, not determined.

Our initial attempts to express *actA* from plasmids typically used to express other genes in *F. johnsoniae* (19–23) were unsuccessful. The plasmids pCP11 (19) and pCP29 (20) carrying *actA* appeared to be lethal to the cells and careful analysis of various genetic constructs implicated the ActA protein, rather than the plasmid DNA itself or the *actA* gene, as the cause of the lethal effect (Table S1). Overexpression of ActA with respect to the other ACIII subunits may be deleterious to the cells. To examine this possibility, we complemented the mutant with expression plasmids modified to reduce the expression level of ActA. We constructed pCP11 plasmids with *actA* placed under the control of the *ompA* promoter (pOmpA) with mutations that have been shown to result in 75, 60, or 42% of wild-type pOmpA activity (pCP11_A75, pCP11_A60, and pCP11_A42, respectively) (24). The specific modifications to reduce pOmpA activity are indicated in Fig. 3A.

Of these three *actA* expression plasmids, pCP11_A75 was apparently lethal, whereas pCP11_A60 and pCP11_A42 were not (Fig. 3B). Furthermore, both A60 and A42 strains (the strains obtained from complementation of Δ*actA* with pCP11_A60 and pCP11_A42, respectively) successfully restored the missing ActA subunit (Fig. 2B). Importantly, the other missing subunit, ActE, was also restored, supporting the idea that incorporation of ActE into the ACIII requires the presence of ActA.

Since the complementation of Δ*actE* with *actE* expressed from the original expression vector pCP29 was unsuccessful, we applied the same strategy to reduce the expression level of ActE. The *actE* gene was placed under the control of unmodified pOmpA (pCP11_E100) or pOmpA with reduced activities (pCP11_E75, pCP11_E60, or pCP11_E42), Fig. 3A. As shown in Fig. 3B, only pCP11_E100 was lethal. All three plasmids with reduced activity of pOmpA were maintained in the cells (strains E75, E60, and E42). However, ActE bands were clearly observed in E42 and E60, whereas in E75 the ActE level was much decreased. The reason for this decrease is unclear and puzzling, especially if the overexpression of these proteins is lethal for the cells. If *actE* is overexpressed from E75, perhaps the high levels of ActE interfere with maturation and, as a result, less ActE is finally incorporated into the membrane-associated ACIII. Alternatively, there might be a strong selection for mutations that decrease *actE* expression, thus reducing toxicity. These could be promoter mutations or mutations that delete or disrupt *actE*. However, we sequenced the entire *actE* gene, along with its promoter sequence, on the pCP11_E75 plasmid isolated from *F. johnsoniae* E75 strain and did not observe such mutations. Another possibility is that suppressor mutations in other regions of the plasmid or in the chromosome reduced ActE expression, allowing growth.

The complementation experiments for ActA and ActE revealed that success in the plasmid-based expression of the individual subunits of ACIII in *F. johnsoniae* requires careful adjustment of the protein expression level. The association of the targeted gene with a strong promoter is likely to be lethal, thus promoters of reduced activities should be applied. In the case of ActA and ActE, the maximal nonlethal gene expression was observed with pOmpA with its activity reduced to 60% (the strains A60 and E60, respectively). Thus, A60 and E60 were used for further functional analyses. ACIII complex from the Strep-tagged E60 strain was purified and the SDS-PAGE analysis revealed proper assembly of the protein complex (Fig. S2).

The growth rates of the complemented mutants (A60 and E60) were examined (Fig. 2A, right). All strains used in this experiment carried either empty pCP11_60% plasmid (WT_p60, Δ*actA*_p60, and Δ*actE*_p60, respectively) or carried pCP11_60% expressing the appropriate protein for complementation (ActA or ActE). This allowed all strains to be grown similarly with erythromycin to compare growth rates. The growth rates for the complemented strains (A60 and E60) were similar to those of the wild-type (WT), and were faster than those of the deletion mutants. WT (Fig. 2A, left, without erythromycin) and WT_p60 (Fig. 2A, right, with erythromycin) showed similar growth curves, but WT_p60 with erythromycin reached a lower maximum optical density at 600 nm (OD_600_). Overall, the results suggest that complementation of the deletion mutants with the appropriate gene restored the functional ACIII complex, thus restoring bacterial growth rate to the WT level.

### EPR-based oximetry to measure ACIII activity in membranes.

The proposed role of ACIII is similar to that of cytochrome *bc_1_*, namely, to transfer electrons from the quinone pool to cytochrome oxidase. In the mitochondrial electron transport chain, this transfer is mediated by a diffusible, water-soluble cytochrome *c*, which accepts electrons from cytochrome *bc_1_* and passes them to cytochrome oxidase. Therefore, a typical approach to measure enzymatic activity of cytochrome *bc_1_* is to follow the rate of reduction of cytochrome *c* in the presence of decylubiquinol (DBH_2_), a water-soluble synthetic quinol analogue. Furthermore, mitochondrial cytochrome *c* accepts electrons not only from its physiological partner, but also from other complexes, such as prokaryotic cytochrome *bc_1_*. For this reason, mitochondrial cytochrome *c* is commonly used in activity assays for diverse respiratory complexes. However, applicability of this assay for ACIII is problematic. Available data indicate that mitochondrial cytochrome *c* is an extremely poor acceptor of electrons from ACIII (3). In our attempts, the kinetic traces of mitochondrial cytochrome *c* reduction by ACIII barely exceeded the background traces of nonenzymatic reduction of cytochrome *c* by DBH_2_. This prevented us from using this method to estimate ACIII activities.

That cytochrome *c* reacts poorly with ACIII might be a consequence of the tight structural association of ACIII and cytochrome *aa_3_* implied from the cryo-EM structures. Since electron transfer between these complexes can occur directly without involvement of a freely diffusible electron carrier, ACIII has likely evolved without the ability to interact with water-soluble proteins such as cytochrome *c* (14).

In view of these considerations, we used an electron paramagnetic resonance (EPR)-based oximetry assay to measure ACIII activity coupled to cytochrome *aa_3_* in native membranes. Electron transfer was triggered by addition of succinate. The measurements were conducted in the absence and presence of cyanide and the difference in kinetic traces recorded under these conditions allowed estimation of cytochrome *aa_3_* contribution to oxygen consumption. This strictly depends on ACIII function, and thus reflects the ability of ACIII to transfer electrons to the oxidase. The observed residual rates of oxygen consumption upon addition of cyanide are most probably an effect of *bd* oxidase activity, which is not blocked by cyanide.

**FIG 4:**
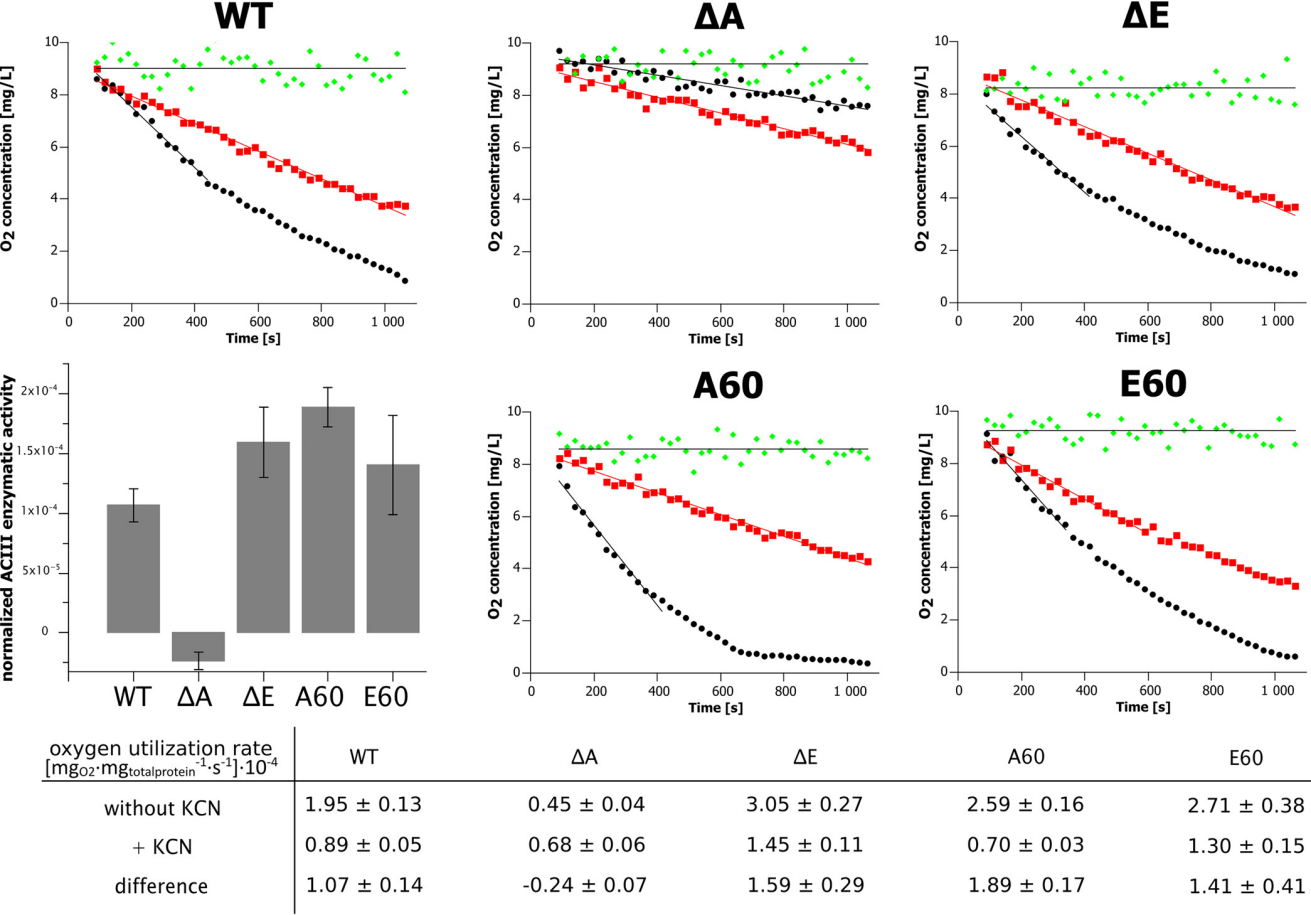
Oxygen consumption rates measured in wild-type and mutant *F. johnsoniae* strains. Green, membranes without succinate addition; black, membranes with succinate addition; red, membranes with the addition of succinate and cyanide to inhibit cytochrome *aa_3_*; ΔA, Δ*actA* strain; ΔE, Δ*actE* strain; A60, Δ*actA* complemented with pCP11_A60; and E60, Δ*actE* complemented with pCP11_E60. The bar graph illustrates the calculated ACIII enzymatic activities in WT and different mutants normalized to total protein content [mg_O2_·mg_total protein_^−1^·s^−1^] and shown in a table below.

The results of the measurements for WT, deletion strains, and complemented mutants, summarized in Fig. 4, reveal that ACIII-cytochrome *aa_3_* was inactive only in the *actA* deletion mutant. All other forms, i.e., the *actE* deletion strain and the complemented A60 and E60 strains, respectively, exhibited clear cyanide-sensitive ACIII-cytochrome *aa_3_* activity. The implications of these findings are discussed below.

### ActE is not essential for electron transfer between ACIII and cytochrome *aa_3_*.

The observation that ACIII-cytochrome *aa*_3_ enzymatic activity in Δ*actE* membranes is comparable to WT (Fig. 4) indicates that ActE is not necessary for electron transfer between ACIII and cytochrome *aa_3_*. Nevertheless, the absence of ActE negatively impacted the growth rate (Fig. 2A), indicating that ActE performs a beneficial function in the cell. The nature of that function is not completely understood.

**FIG 5:**
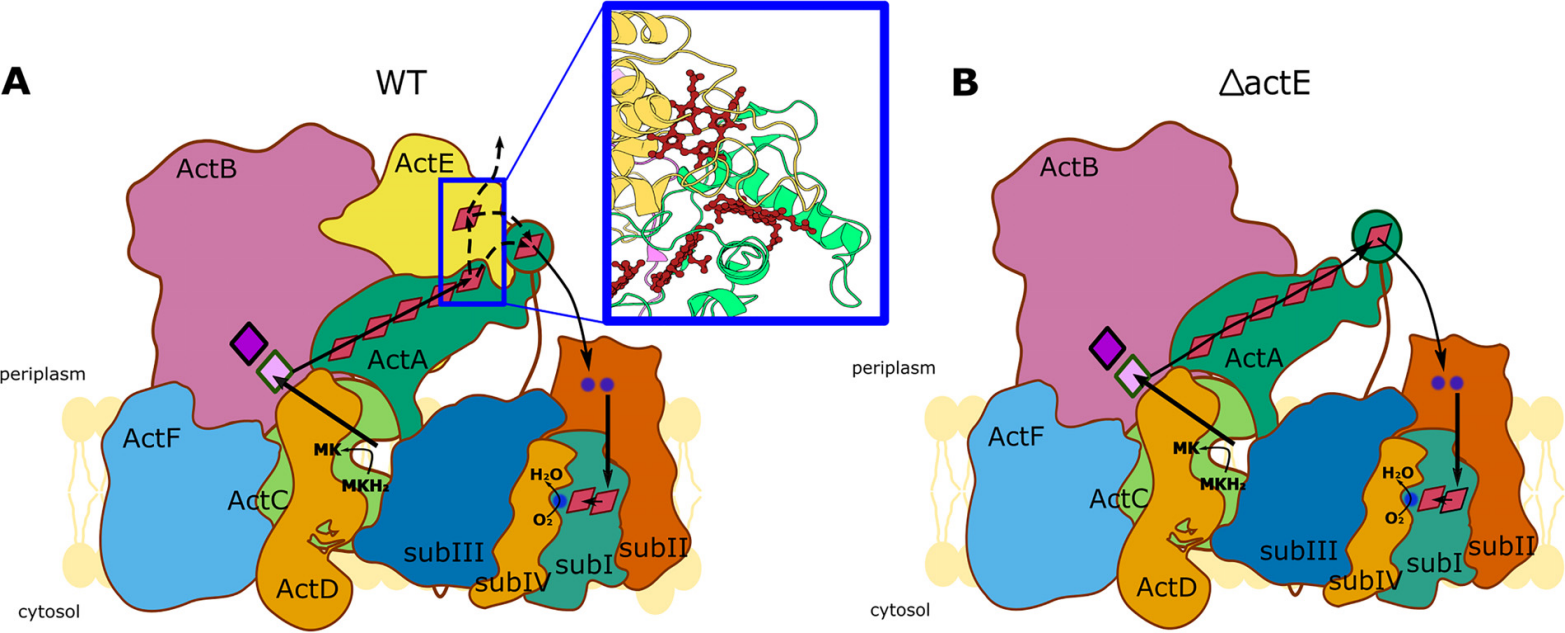
Possible pathways of electron transfer in *F. johnsoniae*. (A) Possible electron transfer paths in ACIII. Dotted arrows indicate distinct hypothetical paths for electron transfer. The inset (blue rectangle) depicts the position of terminal hemes in relation to each other. (B) Predicted electron transfer paths in the Δ*actE* strain.

One possibility is that ActE transfers electrons to another, yet-unidentified electron-accepting partner of ACIII (Fig. 5). This would implicate a split in electron paths at the ActA/ActE interface, a location remote from the MKH_2_ oxidation site. The proposed split within ACIII might resemble that described for Neisseria gonorrhoeae cytochrome oxidase *cbb*_3_. This cytochrome possesses an additional heme *c* at the C-terminal domain of the CcoP subunit. The extra heme is the cofactor at which the split of electron paths takes place. Electrons received from cytochrome *bc_1_* are transferred either through the remaining cofactors of the cytochrome *cbb_3_* or directly to the soluble cytochrome *c_2_* and further to nitrite reductase AniA (25). Similarly to our *actE* deletion strain, when the additional heme *c* of the CcoP subunit is missing, the mutated bacteria exhibit an oxygen consumption rate similar to WT (26). A second possibility arises from the fact that the structure of the mobile globular domain of ActA was not resolved in cryo-EM studies. Thus, its exact shape, docking site(s), range of movement, and distances between interacting cofactors are not known. One cannot exclude that electrons can be transferred to the sixth heme of ActA either from ActE heme or, alternatively, directly from the fifth heme of ActA (Fig. 5). In this scenario, ActE can serve as an electron sink, similar to the dead-end wire of iron-sulfur clusters. In that case, ActE would act as a buffer for electrons, preventing reverse electron flow to the menaquinol-binding site which could result in production of free radicals. Additional studies are needed to determine the exact role of ActE.

Interestingly, the addition of cyanide to the Δ*actA* mutant resulted in an increased residual rate of oxygen consumption (compare the red versus black points in Fig. 4). This was unexpected and clearly different from all other cases, where the addition of cyanide resulted in decreased oxygen consumption (Fig. 4). While the origin of the increased rate of oxygen consumption by the Δ*actA* mutant in the presence of cyanide is not certain, we speculate that cyanide inhibits not only *aa*_3_ oxidase, but also other enzymes that utilize MKH_2_. The only path that is not inhibited by cyanide is through *bd* oxidase. When cyanide is present, more MKH_2_ is available for this enzyme and therefore more oxygen might be reduced in the Δ*actA* mutant under this condition.

### Role of heme-containing subunits in electron transfer.

Our studies revealed that in the Δ*actA* mutant the ACIII-cytochrome *aa_3_* activity was impaired. This is consistent with the role considered for ACIII as the sole source of electrons for cytochrome *aa_3_*. We also show that ActE is not required for electron transfer between *F. johnsoniae* ACIII and cytochrome *aa_3_*. In the Δ*actE* mutant, the electron transfer from ACIII to cytochrome *aa_3_* may occur directly from ActA, presumably because of the presence of the N-terminal sixth heme of the ActA mobile domain.

Interestingly, in *R. marinus* this domain is missing, but the direct electron transfer to the terminal oxidase is still possible, most likely because the *R. marinus* cytochrome *caa_3_* contains an additional heme *c* (7). We note that the lack of the sixth heme in *R. marinus* ActA might make ActE essential for electron transfer to cytochrome *caa_3_* in this bacterium, in contrast to the situation for *F. johnsoniae.* In view of these considerations the organization of heme cofactor chain proposed for *F. johnsoniae* might not be universal for all species.

Bioinformatic analysis revealed organisms in which the ActE subunit is missing from the gene cluster coding for other subunits of ACIII complex (9). In Pelobacter carbinolicus, the lack of ActE may be functionally compensated by other protein(s) containing a monoheme cytochrome *c* domain. However, in Sphaerobacter thermophilus and *Geobacter* sp. strain M21, ActE seems to be lacking without any substitution in the ACIII gene cluster. These observations reveal that ActE is not evolutionary conserved, which is in line with our results showing it to be dispensable for maintaining ACIII activity in *F. johnsoniae*.

*R. marinus* ActE was shown to react with two structurally different electron acceptors: high potential iron-sulfur protein (HiPIP) and soluble cytochrome *c* (3, 27, 28). In view of our finding that *F. johnsoniae* ActE is dispensable for electron transfer to cytochrome *aa_3_*, yet beneficial for cell growth, we speculate that it might also react with more than one electron acceptor. In this way, ActE might direct electrons outside the ACIII-cytochrome *aa_3_* supercomplex, offering an alternative path to oxidize the menaquinone pool. Clearly the concept of multiplicity of electron paths that are linked functionally by ACIII is intriguing and worth further exploration. We anticipate that the genetic system described here, in combination with spectroscopic studies, will allow us to address these and other mechanistic issues of ACIII and its macromolecular interactions.

## MATERIALS AND METHODS

### Bacterial strains, plasmids, and primers.

The donor strain of Escherichia coli used for conjugative plasmid transfer was E. coli TOP10 (IBA Lifesciences) or HB101. E. coli strain HB101 carrying the pRK2013 plasmid (29) was used as a helper for triparental conjugation. All primers and plasmids used in this study are listed in Table S2 and Table S3, respectively, in the supplemental material. Antibiotics were used at the indicated concentrations when needed: ampicillin 100 μg ml^−1^; erythromycin 100 μg ml^−1^, kanamycin 50 μg ml^−1^.

### Conjugative gene transfer into *F. johnsoniae*.

E. coli HB101 containing pYT354-derivative suicide vectors or E. coli TOP10 containing pCP vectors, E. coli containing pRK2013, and *F. johnsoniae* were grown overnight, harvested (1 ml each, 1,700 × *g*, 4 min) and washed once with charcoal-yeast extract (CYE) medium. The cell pellets were pooled, suspended in 100 μl of CYE medium, and spotted on CYE-agar plates. Following overnight incubation, the cells were scraped off the agar, diluted in 2 ml CYE medium, and plated on CYE-agar containing erythromycin. Plates were incubated for 48 h at 30°C (18).

### Deletion of *F. johnsoniae actA*.

An approximately 2-kbp fragment upstream of Fjoh_1634 was amplified using primers 1 (introducing a NotI site) and 2 (introducing a BamHI site). The fragment was digested with NotI and BamHI and ligated into pYT354, which had been digested with the same enzymes, to generate pYT354.12. An approximately 2-kbp fragment downstream of Fjoh_1634 was amplified using primers 3 (introducing a BamHI site) and 4 (introducing a KpnI site). The fragment was digested with BamHI and KpnI and ligated into pYT354.12, which had been digested with the same enzymes, to generate pYTactAfl. This construct was introduced into *F. johnsoniae* WT by conjugation. The following procedures were performed as in reference 18. Colonies were screened by PCR using primers 5S and 11S to identify the *actA* deletion mutant (Δ*actA*). Three independent experiments resulted in three colonies with deletion of the *actA* gene, which was additionally confirmed by PCR amplification and sequencing of a DNA fragment amplified with 9S and 10S primers. The Δ*actA* mutant was cultured without antibiotics.

### Deletion of *F. johnsoniae actE*.

The *actE* gene was deleted as described above for *actA*, except that primers 10, 11, 12, and 13 were used to construct the deletion plasmid pYTactEfl. Colonies were screened by PCR using primers 15S and 16S to identify the *actE* deletion mutants (Δ*actE*). These were additionally confirmed by PCR amplification and sequencing of a DNA fragment amplified with primers 14S and 19S. The Δ*actE* mutant was cultured without antibiotics.

### *actA* gene complementation.

The *ompA* promoter was amplified from pYT313 and introduced into the MCS of pCP11 using primers introducing BamHI and NotI sites. During PCR amplification, the following desired promoter mutations were introduced (24): (i) deletion of _-53_TTTTTT_-48_ upstream of the −33 region of the promoter (approximately 42% native promoter activity, pCP11_42%) (primers 46 and 47); (ii) substitution in the −33 region of the promoter, _-35_TTG_-33_ -> TGG (approximately 60% native promoter activity, pCP11_60%) (primers 48 and 47); (iii) substitution in the −7 region of the *ompA* promoter, _-10_TTT_-8_ -> CTT (approximately 75% native promoter activity, pCP11_75%) (primers 49 and 50; and (iv) unchanged *ompA* promoter sequence (100% native promoter activity, pCP11_100%) (primers 49 and 47). Using primers 25 (introducing a NotI site) and 8 (introducing an XmaI site), we amplified and cloned the *actA* gene into pCP11 vectors with different o*mpA* promoter versions to generate pCP11_A42, pCP11_A60, and pCP11_A75, respectively. The correct sequence of the constructs was confirmed by sequencing and plasmids were introduced into *F. johnsoniae* Δ*actA* by conjugation. After conjugation, bacteria were cultured with 100 μg ml^−1^ erythromycin. Plasmids extracted directly from *F. johnsoniae* were sequenced.

### *actE* gene complementation.

Using primers 26 (introducing a NotI site) and 27 (introducing an XmaI site), we amplified and cloned the *actE* gene into pCP11 vectors with different *ompA* promoter versions to generate pCP11_E42, pCP11_E60, pCP11_E75, and pCP11_E100, respectively.

### Growth rate measurements.

Overnight cultures of bacterial strains were diluted to OD_600_ = 0.1 in 20 ml of CYE medium supplemented with the appropriate antibiotics where needed. Cells were grown at 30°C in an orbital shaker at 160 rpm. The OD_600_ was measured every hour.

### Membrane preparation.

Cells were grown overnight and collected by centrifugation (6,000 × *g* for 30 min). The cell pellet from a 5-liter culture was resuspended in 100 ml of 20 mM Tris (pH 8), 50 mM NaCl, and 1 mM EDTA in the presence of protease inhibitors (17 mg phenylmethylsulfonyl fluoride, 13 mg 6-aminocaproic acid, and 15 mg benzamidine hydrochloride). The suspension was passed three times through a French press to disrupt the cells. The cell extract was centrifuged at 14,000 × *g* for 30 min and then 180,000 × *g* for 1.5 h. The membrane pellet was resuspended in 20 mM Tris (pH 8), 100 mM NaCl, and 1 mM EDTA to a final concentration of about 50 mg/ml of total protein. Total protein concentration was determined using a Pierce BCA protein assay kit (Thermo-Scientific). The membrane samples were analyzed using SDS-PAGE using 4 to 20% precast gels (Bio-Rad). Heme *c* staining was accomplished using 3,3′,5,5′-tetramethyl benzidine (TMBZ) (30).

### Measurement of oxygen consumption.

Oxygen consumption was measured using EPR spectroscopy on a Bruker ElexSys E580 spectrometer by determining the oxygen-dependent amplitudes of the superhyperfine structure of CTPO spin label (k parameter), as described in reference 31. A calibration curve relating spectral properties of CTPO to oxygen concentration, i.e., [O_2_] = f(k), was prepared using buffers containing different proportions of air to argon. Gases were mixed with the WITT gas mixer and oxygen concentrations in each control samples were determined using the Mettler Toledo InLab OptiOx sensor. Oxygen consumption by the bacterial membranes was measured in 20 mM Tris (pH 8), 100 mM NaCl, and 1 mM EDTA. Each sample contained 170 μl of the membrane suspension, 4 μl CTPO (final concentration 100 μM) and 176 μl of the buffer containing KCN (final concentration 5 mM) or without KCN. The reaction was started by addition of 50 μl of sodium succinate (final concentration 6.25 mM). After addition of succinate, the EPR cuvettes were inserted into the resonator and the spectra of CTPO were automatically recorded in 40-s intervals. EPR spectrometer settings were as follows: microwave power 6.416 mW (15 dB), modulation amplitude/frequency 0.08 G/100 kHz, sweep width/time 4 G/10.5 s, time constant 20.48 ms, conversion time 10.24 ms, resonator TM_110_ and the gas-tight quartz flat cuvette containing 0.2 ml of a sample. All spectra were analyzed using a custom-written computer program (Eleana 4.0 Beta).

### Construction of Strep-tagged ActE.

The *actE* gene was amplified using primers 26 (introducing a NotI site) and 30 (introducing a Strep tag). The amplified product was then used as a template to obtain *actE* gene with a Strep tag using primers 26 and 31 (introducing a Strep tag and an XmaI site). The amplicon was cloned into pCP11_60% vector to generate pCP11_EST60.

### Protein purification.

A membrane pellet from Strep-tagged E60 was resuspended in 20 mM Tris (pH 8), 100 mM NaCl, 1 mM EDTA to a final concentration of 50 mg/ml of total protein, followed by 1.5 g of DDM (n-dodecyl β-d-maltoside, Anatrace) per 1 g of total protein. After 1 h of incubation, the sample was ultracentrifuged for 30 min at 180,000 × *g* and filtered with 0.45-μm syringe filter. The sample was loaded on the Strep-Tactin column (IBA Lifesciences) preequilibrated with 20 mM Tris (pH 8), 100 mM NaCl, 1 mM EDTA, 0.05% DDM buffer. The subsequent steps were performed according to the manufacturer’s protocol (IBA Lifesciences). The total protein concentration was determined using a Pierce BCA protein assay kit. Purified protein samples were analyzed using SDS-PAGE using 4 to 20% precast gels (Bio-Rad).

### Data sharing.

All materials and data generated in this work are available upon request from corresponding authors.
