# Supplementary material for: The Monoheme c Subunit of Respiratory Alternative Complex III Is Not Essential for Electron Transfer to Cytochrome aa3 in Flavobacterium johnsoniae
Source: Microbiol Spectr. 2021 Jun 30;9(1):10.1128/spectrum.00135-21. doi: 10.1128/spectrum.00135-21 (PMC8552683; doi:10.1128/spectrum.00135-21)
Supplement: SUPPLEMENTAL FILE 1 — Supplemental text, Fig. S1 and S2, and Tables S1 to S3. Download SPECTRUM00135-21_Supp_1_seq9.pdf, PDF file, 0.388 MB [file spectrum00135-21_supp_1_seq9.pdf]

Supplemental Material for

The monoheme *c* subunit of respiratory alternative complex III is not essential for electron transfer to cytochrome *aa*$_3$ in *Flavobacterium johnsoniae*

Katarzyna Lorencik, Robert Ekiert, Yongtao Zhu, Mark J. McBride, Robert B. Gennis, Marcin Sarewicz, Artur Osyczka

actA and actE gene deletion

The *actA* flanking regions were PCR-amplified from the *F. johnsoniae* genomic DNA with 1, 2, 3, 4 primers (Table S2) and cloned into pYT354 vector generating pYTactAfl (Fig. S1), conferring erythromycin resistance and sucrose sensitivity. The construct was introduced into *F. johnsoniae* WT by conjugation. Similar strategy was employed for *actE* deletion using primers 10, 11, 12 and 13 to make pYTactEfl construct.

In the first recombination event, integration of the plasmid into bacterial genome occurs by recombination of either the region upstream or downstream of the gene to be deleted (Fig. S1A). After this process, the entire plasmid is integrated into the chromosome with the position dependent on the recombination site (Fig. S1B). Subsequently, bacteria are cultured without antibiotic selection to allow second recombination event, in which the plasmid is lost from the genome. Regardless of the first recombination site, the second recombination results in either deletion or WT genotype (Fig. S1C). Cells that underwent second recombination are selected by culture in the presence of sucrose (Fig. S1D). Only bacteria that have lost the plasmid are able to grow on sucrose.

From 52 tested colonies after second recombination events, we obtained three colonies with *actA* gene deletion. The remaining 49 colonies were WT. For *actE* deletion the efficiency was two colonies out of 51.

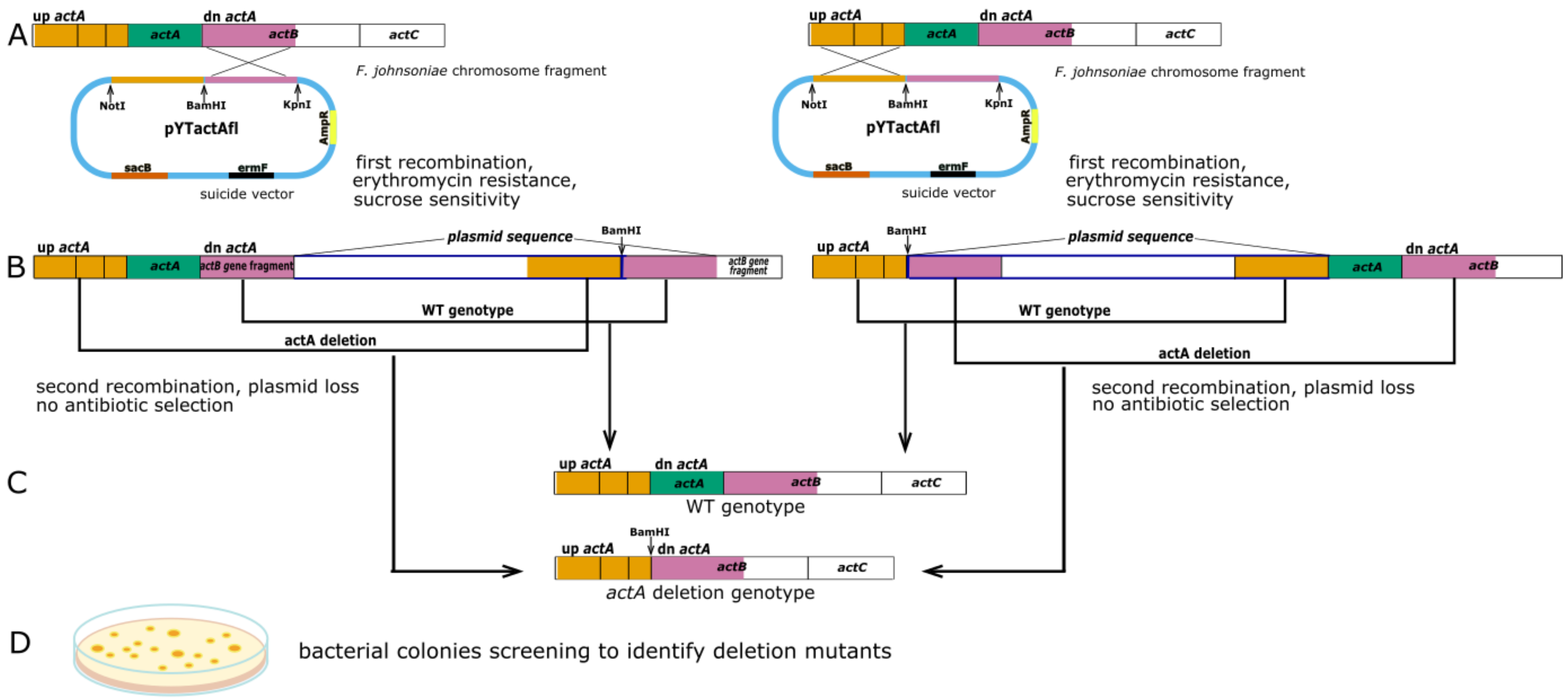

Figure S1. ***Deletion of actA gene.*** *Green rectangle – actA gene, light orange – sequence upstream (up) actA, pink – sequence downstream (dn) actA, orange – sucrose sensitivity gene for F. johnsoniae, black – erythromycin resistance gene for F. johnsoniae, yellow – ampicillin resistance gene for E. coli. A. Recombination between upstream/ downstream region on the plasmid and in the bacterial genome. B. Fragment of bacterial chromosome with integrated plasmid. Horizontal connections depict possible ways of second recombination. C. Resulting fragment of chromosome after second recombination. D. Last step of the gene deletion protocol: PCR-based screening of numerous bacterial colonies to identify the deletion mutants.*

Construction of complementation vectors

To complement the deleted genes we chose standard plasmid typically used for gene reintroduction in *Flavobacterium johnsoniae* (pCP11). The pCP11 plasmid does not contain any gene promoter preceding multicloning site, thus we introduced *actA* gene with its 200 nucleotides upstream sequence (between Fjoh_1633 and Fjoh_1634). Introduction of such plasmid into *F. johnsoniae* WT was unsuccessful, no colonies were obtained. Interestingly, introduction of empty pCP11 plasmid resulted in multiple colonies. In order to test whether pCP11_*actA* uptake is unfeasible due to its length we tested several other constructs based on pCP29 vector, which contains its own gene promoter. Similarly to pCP11, bacteria transformed with empty pCP29 were able to grow, but pCP29 with *actA* was lethal, regardless of the promoter used. Additionally, *actA* was cloned in inverted orientation (3'-5') to test if the plasmid with this length is taken up with as good effectiveness as empty pCP29 vector. Such construct sustained bacterial growth. Simultaneously, the same experiments were performed with Δ*actA* mutant giving identical results, which suggests that ActA protein overexpression is toxic for the cells. Furthermore, ability of Δ*actA* strain to grow with empty plasmids suggests that this mutant is feasible to further genetic modifications.

Table S1. Genetic constructs used to identify the reason of lethality of the plasmid uptake.

**Bacterial strain**	**Plasmid**	**Promoter**	**Gene**	**Growth +/–**
WT	pCP11	—	—	**+**
WT	pCP11_A_p	Native (from genome)	*actA*	**–**
WT	pCP29	From plasmid	—	**+**
WT	pCP29_A	From plasmid	*actA*	**–**
WT	pCP29_A_inv	From plasmid	*actA* inverted orientation	**+**
WT	pCP29_A_p	Native	*actA*	**–**
ΔactA	pCP11	—	—	**+**
ΔactA	pCP11_A_p	Native	*actA*	**–**
ΔactA	pCP29	From plasmid	—	**+**
ΔactA	pCP29_A	From plasmid	*actA*	**–**
ΔactA	pCP29_A_inv	From plasmid	*actA* inverted orientation*	**+**
ΔactA	pCP29_A_p	Native	*actA*	**–**

+ colonies were obtained
– construct is lethal

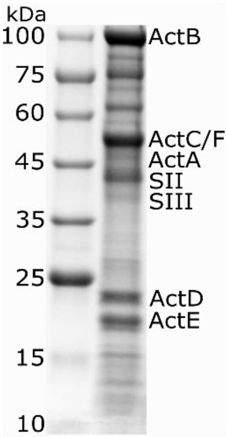

Figure S2. SDS-PAGE analysis of purified ACIII-cytochrome aa_3 supercomplex. Proteins were isolated from Strep-tagged E60 strain using affinity chromatography and separated on 4-20% gradient gel stained with Coomassie Brilliant Blue. m – protein marker, the individual subunits of ACIII-cytochrome aa_3 supercomplex are marked on the right.

Table S2. Primers used in this study.

Primer	Sequence 5'-> 3'
1	TTGCGGCCGCTATTAATGCAATTTTAAG
2	CGCGGATCCATAGTATAATAATTATCTAC
3	CGCGGATCCTAATCGAATTATTAAGATTTTAATATATATATAC
4	GGGGTACCTTTACGACCGTATCCAAC
5S	TTATCTCGCTTTTTTTATTC
7	CGCGGATCCAATGTGATGGTTTTTATGC
8	CCCCGGGCGATTAATAGTGGCATTTACC
9S	CCTAACCCCCAAAGGAAGGC
10S	GGATTGTTTGTTTCTTTGTCTTTAAAAATAG
10	TTGCGGCCGCGTAAAATTATTAATTAAGAAAC
11	CGCGGATCCAGCTACAATTAATTCTTTTC
11S	CATAAGGGATAGACTTGTGC
12	CGCGGATCCTTGTTGAACAAACTGATCG
13	CGGGGTACCTCTTGTCCAGCATATCTAGC
14S	ATCACGTAAAAGTTAGAAACAC
15S	GTTGCTGGAGCGGTTTTCTC
16S	TGAGCATCTGCTGCTAGTAT
19S	GGGTCTGACATATCAACTCC
21	CGGGGTACCAATGTGATGGTTTTTATGC
22F	CGGGGTACCATGAAAAAGGTGGGTAACC
22	CATGCATGCTTAATAGTGGCATTTACCGC
23	CATGCATGCATGAAAAAGGTGGGTAACC
24	CGGGGTACCTTAATAGTGGCATTTACCGC
25	ATTTGCGGCCGCATGAAAAAGGTGGGTAACC
26	ATTTGCGGCCGCTTGTAGCTATGAAAAGG
27	CCCCCCGGGCAATTATAATTGGCTTTTTAG
30	GAACTGCGGGTGGCTCCATAATTGGCTTTTTAG
31	GCAGTTCGAGAAATAATCGCCCGGGG
46	CGCGGATCCTTAACATTTGATTTTGTA
47	ATTTGCGGCCGCTACAATTTAGTTAATTACAAG
48	CGCGGATCCTTTTTGTAGGTTTTTTTTAACATTTGATTTGGTATTTAAA
49	CGCGGATCCTTTTTGTAGGTTTTTTTTAAC
50	ATTTGCGGCCGCAATTACAAGCAAAGGTAACACC

Table S3. Plasmids used in this study

Plasmid	Description*	Source or reference
pYT313	Suicide vector carrying *sacB;* Amp (Erm)	(1)
pYT354	Suicide vector carrying *sacB* with MCS of pBC SK+; Amp (Erm)	(1)
pYTactAfl	*actA* deletion construct, 2 kbp fragment upstream of *actA* amplified using primers 1 and 2 and 2 kbp fragment downstream of *actA* amplified using primers 3 and 4 and cloned into *Not*I and *Kpn*I sites of pYT354; Amp (Erm)	This study
pYTactEfl	*actE* deletion construct, 2 kbp fragment upstream of *actE* amplified using primers 10 and 11 and 2 kbp fragment downstream of *actE* amplified using primers 12 and 13 and cloned into *Not*I and *Kpn*I sites of pYT354; Amp (Erm)	This study
pRK2013	Helper plasmid for triparental conjugation; Km	(2)
pCP11	*E. coli-F. johnsoniae* shuttle plasmid; Amp (Erm)	(3)
pCP11_A_p	*actA* expression construct for *F. johnsoniae*; *actA* with its 219 bp upstream region was amplified using primers 7 and 8 and cloned into *BamH*I and *Xma*I sites of pCP11; Amp (Erm)	This study
pCP11_42%	Expression vector for *F. johnsoniae* with ~42% activity of *ompA* promoter; *ompA* promoter amplified with primers 46 and 47 from pYT313 and cloned into *BamH*I and *Not*I sites of pCP11; Amp (Erm)	This study
pCP11_60%	Expression vector for *F. johnsoniae* with ~60% activity of *ompA* promoter; *ompA* promoter amplified with primers 47 and 48 from pYT313 and cloned into *BamH*I and *Not*I sites of pCP11; Amp (Erm)	This study
pCP11_75%	Expression vector for *F. johnsoniae* with ~75% activity of *ompA* promoter; *ompA* promoter amplified with primers 49 and 50 from pYT313 and cloned into *BamH*I and *Not*I sites of pCP11; Amp (Erm)	This study
pCP11_100%	Expression vector for *F. johnsoniae* with unmodified *ompA* promoter; *ompA* promoter amplified with primers 47 and 49 from pYT313 and cloned into *BamH*I and *Not*I sites of pCP11; Amp (Erm)	This study
pCP11_A42	*actA* expression construct for *F. johnsoniae* with ~42% activity of *ompA* promoter; *actA* was amplified using primers 25 and 8 and cloned into *Not*I and *Xma*I sites of pCP11_42%; Amp (Erm)	This study
pCP11_A60	*actA* expression construct for *F. johnsoniae* with ~60% activity of *ompA* promoter; *actA* was amplified using primers 25 and 8 and cloned into *Not*I and *Xma*I sites of pCP11_60%; Amp (Erm)	This study
pCP11_A75	*actA* expression construct for *F. johnsoniae* with ~75% activity of *ompA* promoter; *actA* was amplified using primers 25 and 8 and cloned into *Not*I and *Xma*I sites of pCP11_75%; Amp (Erm)	This study
pCP11_E42	*actE* expression construct for *F. johnsoniae* with ~42% activity of *ompA* promoter; *actA* was amplified using primers 26 and 27 and cloned into *Not*I and *Xma*I sites of pCP11_42%; Amp (Erm)	This study
pCP11_E60	*actE* expression construct for *F. johnsoniae* with ~60% activity of *ompA* promoter; *actA* was amplified using primers 26 and 27 and cloned into *Not*I and *Xma*I sites of pCP11_60%; Amp (Erm)	This study

pCP11_E75	*actE* expression construct for *F. johnsoniae* with ~75% activity of *ompA* promoter; *actE* was amplified using primers 26 and 27 and cloned into *Not*I and *Xma*I sites of pCP11_75%; Amp (Erm)	This study
pCP11_E100	*actE* expression construct for *F. johnsoniae* with unmodified *ompA* promoter; *actE* was amplified using primers 26 and 27 and cloned into *Not*I and *Xma*I sites of pCP11_100%; Amp (Erm)	This study
pCP11_EST60	Strep-tagged *actE* expression construct for *F. johnsoniae* with ~60% activity of *ompA* promoter; *actE* was amplified using primers 26 and 31 (introducing Strep-tag) and cloned into *Not*I and *Xma*I sites of pCP11_60%; Amp (Erm)	This study
pCP29	*E. coli-F. johnsoniae* shuttle plasmid; Amp (Cm, Erm)	(4)
pCP29_A	*actA* expression construct for *F. johnsoniae*; *actA* was amplified using primers 22F and 22 and cloned into *Kpn*I and *Sph*I sites of pCP29; Amp (Erm)	This study
pCP29_A_p	*actA* expression construct for *F. johnsoniae*; *actA* with its 219 bp upstream region was amplified using primers 21 and 22 and cloned into *Kpn*I and *Sph*I sites of pCP29; Amp (Erm)	This study
pCP29_A_inv	*actA* expression construct for *F. johnsoniae*; *actA* was amplified using primers 23 and 24 and cloned in inverted orientation into *Sph*I and *Kpn*I sites of pCP29; Amp (Erm)	This study
pCP29_E	*actE* expression construct for *F. johnsoniae*; *actE* was amplified using primers 14 and 15 and cloned into *Sph*I and *Kpn*I sites of pCP29; Amp (Erm)	This study

*Antibiotic resistance phenotypes expressed in *E. coli*: ampicillin, Amp; kanamycin, Km. Antibiotic resistance phenotypes expressed in *F. johnsoniae* (given in parentheses)*:* chloramphenicol, Cm; erythromycin, Erm.

References

1. Zhu Y, Thomas F, Larocque R, Li N, Duffieux D, Cladière L, Souchaud F, Michel G, McBride MJ. 2017. Genetic analyses unravel the crucial role of a horizontally acquired alginate lyase for brown algal biomass degradation by *Zobellia galactanivorans*. Environ Microbiol 19:2164–2181.

2. Figurski DH, Helinski DR. 1979. Replication of an origin-containing derivative of plasmid RK2 dependent on a plasmid function provided in *trans*. Proc Nati Acad Sc 76:1648–1652.

3. McBride MJ, Kempf MJ. 1996. Development of techniques for the genetic manipulation of the gliding bacterium *Cytophaga johnsonae*. J Bacteriol 178:583–590.

4. Kempf MJ, McBride MJ. 2000. Transposon insertions in the *Flavobacterium johnsoniae ftsX* gene disrupt gliding motility and cell division. J Bacteriol 182:1671–1679.
